# Phase I dose escalation and pharmacokinetic study of pluronic polymer-bound doxorubicin (SP1049C) in patients with advanced cancer

**DOI:** 10.1038/sj.bjc.6601856

**Published:** 2004-05-04

**Authors:** S Danson, D Ferry, V Alakhov, J Margison, D Kerr, D Jowle, M Brampton, G Halbert, M Ranson

**Affiliations:** 1 Department of Medical Oncology, Christie Hospital NHS Trust, Wilmslow Road, Withington, Manchester M20 4BX, UK; 2 Queen Elizabeth University Hospital Trust, Birmingham B15 2TH, UK; 3 Supratek Pharma Inc., 531 Blvd des Prairies, Build. 18, Laval, Quebec, Canada H7B 1B7; 4 Cancer Research UK, PO Box 123, London WC2A 3PX, UK; 5 Cancer Research UK Formulation Unit, Department of Pharmaceutical Studies, University of Strathclyde, Royal College Building, 204 George Street, Glasgow G1 1XW, UK

**Keywords:** SP1049C, doxorubicin, polymer, pharmacokinetics, phase I

## Abstract

SP1049C is a novel anticancer agent containing doxorubicin and two nonionic pluronic block copolymers. In preclinical studies, SP1049C demonstrated increased efficacy compared to doxorubicin. The objectives of this first phase I study were to determine the toxicity profile, dose-limiting toxicity, maximum tolerated dose and pharmacokinetic profile of SP1049C, and to document any antitumour activity. The starting dose was 5 mg m^−2^ (doxorubicin content) as an intravenous infusion once every 3 weeks for up to six cycles. A total of 26 patients received 78 courses at seven dose levels. The dose-limiting toxicity was myelosuppression and DLT was reached at 90 mg m^−2^. The maximum tolerated dose was 70 mg m^−2^ and is recommended for future trials. The pharmacokinetic profile of SP1049C showed a slower clearance than has been reported for conventional doxorubicin. Evidence of antitumour activity was seen in some patients with advanced resistant solid tumours. Phase II trials with this agent are now warranted to further define its antitumour activity and safety profile.

Doxorubicin is one of the most widely used and active anticancer agents. Its clinical usefulness is limited by inherent or induced drug resistance and by toxicity considerations. Several approaches have been used to try and improve the therapeutic efficacy and toxicity profile of doxorubicin including the development of anthracycline analogues and the incorporation of anthracyclines into drug delivery vehicles, such as liposomes and polymeric drug conjugates (Ranson *et al*, 1997; Vasey *et al*, 1999). SP1049C represents a novel approach in which doxorubicin is formulated with two nonionic pluronic block copolymers, Pluronic L61 and Pluronic F127 (Figure 1

**Figure 1:**
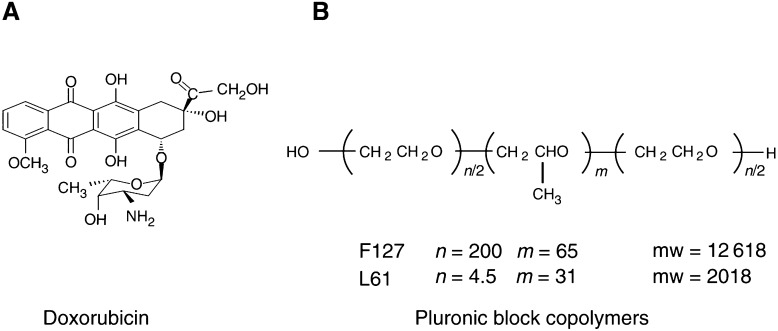
Structures of (**A**) doxorubicin (**B**) block copolymers.

) (Collet, 2003). Doxorubicin is noncovalently incorporated into micelles with the block copolymers, and in standard XTT *in vitro* cytotoxicity assays (Venne *et al*, 1996), SP1049C exhibits greater efficacy than doxorubicin against a variety of drug-resistant tumour cell lines. This improved efficacy of SP1049C *in vitro* appears to be due to increase in cellular drug influx, inhibition of energy-dependent drug efflux and changes in intracellular drug trafficking (Venne *et al*, 1996). Compared to doxorubicin, SP1049C demonstrated superior antitumour activity *in vivo* in multiple animal tumour models and activity in doxorubicin-resistant settings (Batrakova *et al*, 1996; Alakhov *et al*, 1999). The plasma pharmacokinetics and tissue distribution of doxorubicin when administered as SP1049C have been studied in normal and tumour-bearing mice. Increased tissue area under the curves (AUC) were seen in tumour and brain with SP1049C compared to those seen with equivalent doses of free doxorubicin. Area under the curves and maximal tissue concentrations were similar for SP1049C and free doxorubicin in liver, kidney, heart, lung and plasma. SP1049C exhibited similar toxicology to conventional doxorubicin in toxicology studies in mice and rats (Alakhov *et al*, 1999).

This is the first clinical trial of SP1049C. The primary objectives were to determine the toxicity profile, dose-limiting toxicity (DLT), maximum-tolerated dose (MTD) and pharmacokinetic profile of SP1049C. The secondary objective was to evaluate the antitumour effects of SP1049C in patients with advanced cancer.

## PATIENTS AND METHODS

### Inclusion and exclusion criteria

Patients with histologically proven cancer refractory to conventional treatment or for which no suitable conventional therapy existed were considered for entry into this study. Patients were required to be ⩾18 years of age with a Karnofsky Performance Status of 80 or greater and an expected survival of 12 weeks or more. They needed adequate haematological and biochemical function with haemoglobin ⩾10.0 g dl^−1^, total white blood cells (WBC) ⩾3.0 × 10^9^ l^−1^, absolute neutrophil count (ANC) ⩾2.0 × 10^9^ l^−1^ and platelets ⩾100 × 10^9^ l^−1^, and serum creatinine ⩽130 *μ*mol l^−1^ (if >130 *μ*mol l^−1^ then creatinine clearance must be >50 ml min^−1^), serum bilirubin ⩽20 *μ*mol l^−1^, serum AST or ALT ⩽2 × upper limit of the normal. A left ventricular ejection fraction (LVEF) ⩾55% by multinucleated gated angiography (MUGA) was also required.

Patients were excluded if they fulfilled any of the following: major thoracic or abdominal surgery in the preceding 4 weeks; chemotherapeutic agents, experimental drugs or radiation therapy (other than local radiotherapy not involving bone marrow reserves) within the 4 weeks prior to therapy; mitomycin C or nitrosoureas within the previous 6 weeks; any persistent toxic manifestations of previous treatments (except alopecia) must have resolved, or be no greater than Grade 1 and have been stable for over 4 weeks prior to entry; active uncontrolled infection, or other significant nonmalignant intercurrent illness; pregnant or lactating women (negative serum pregnancy test prior to enrolment in those women with child-bearing potential and all patients were required to use medically approved contraceptive precautions during the study and for 4 weeks afterwards); known or suggested central nervous system metastases; known hypersensitivity to anthracycline therapy; prior radiotherapy to more than one-third of haemopoietic sites; clinically significant hepatic disease other than involvement by tumour; history of cardiac disease with New York State Heart Association Class II level or greater or myocardial infarction within the past year; any other condition which in the Investigator's opinion would not make the patient a good candidate for the study.

The study was conducted under the auspices of the Cancer Research Campaign (now Cancer Research UK) in accordance with the principles of the International Conference on Harmonisation of Good Clinical Practice (ICH-GCP) guidelines and the Declaration of Helsinki. The trial protocol was approved by the Local Research Ethics Committee covering each trial centre and all patients gave informed written consent.

### Treatment

SP1049C carrier solution was supplied by Supratek Pharma Inc. (Canada) in 100 ml vials containing Pluronic L61 (0.25% w v^−1^) and Pluronic F127 (2% w v^−1^) in 0.9% sodium chloride for injection. Doxorubicin hydrochloride from Pharmacia Upjohn was provided in 10 and 50 mg vials. In total, 25 ml (or 5 ml for smaller doses) of SP1049C carrier solution for intravenous (i.v.) injection was used to reconstitute 50 mg (or 10 mg for smaller doses) vials of doxorubicin hydrochloride under aseptic conditions to provide a solution of doxorubicin hydrochloride 2.0 mg ml^−1^ in carrier solution. The reconstituted SP1049C contained 2.0 mg ml^−1^ doxorubicin hydrochloride, 2.5 mg ml^−1^ Pluronic L61 and 20 mg ml^−1^ Pluronic F127 in 0.9% sodium chloride. The pluronic carrier solution and reconstituted SP1049C were stored at 4°C. Reconstituted SP1049C stored at 4°C was used within 24 h of reconstitution.

SP1049C was administered via a peripheral i.v. cannula at a rate of 2 ml min^−1^. The starting dose (expressed as doxorubicin content) was 5 mg m^−2^, which was approximately a tenth of the MTD in the mouse and rat toxicology studies. An i.v. infusion of SP1049C was given once every 3 weeks for a maximum of six cycles.

### Toxicity and response evaluation

Pretreatment evaluation included a complete medical history and physical examination as well as full blood count, biochemical profile, routine urinalysis, 12-lead electrocardiogram (ECG) and MUGA. Female subjects with child-bearing potential also underwent a pregnancy test. A baseline chest radiograph and appropriate radiology to evaluate the disease were performed. Tumour markers were also measured if applicable.

Safety was evaluated by recording signs and symptoms during the study period, monitoring vital signs, urinalysis, ECG, MUGA (at baseline, after every two courses and at withdrawal) and laboratory tests (haematological and biochemical). All toxicities were graded according to the National Cancer Institute Common Toxicity Criteria Version 2 (NCI, 1998) and were assessed weekly and at the end of each cycle of SP1049C. Dose-limiting toxicities were defined as any of the following events: (1) two out of six (or ⩾30%) patients experienced Grade 4 neutropenia lasting >5 days or if associated with fever, (2) two out of six (or ⩾30%) patients experienced Grade 4 thrombocytopenia, (3) two out of six (or ⩾30%) patients had Grade 3 or Grade 4 nonhaematological toxicity (excluding Grade 3 nausea and vomiting in patients who have not received optimal treatment with antiemetics), (4) drug-related death. The MTD was defined as the dose level of SP1049C below that at which 30% or more of the patient population experience DLT due to SP1049C. The MTD was to be the dose recommended for phase II studies. Patients were to be followed up until 4 weeks after the last administration of SP1049C. Response was assessed using WHO response criteria (WHO, 1979) at baseline, after every two cycles and after withdrawal. The duration of response was defined as the interval from the date of first treatment to the date of documented disease progression.

### Dose escalation

It was planned that cohorts of patients would be treated with SP1049C at 5, 10, 20, 35, 50, 70 and 90 mg m^−2^ (expressed as doxorubicin content). At the first three dose levels (5, 10 and 20 mg m^−2^) if no toxicity greater than Grade 1 was encountered during the first cycle of SP1049C in the first patient at that level, then subsequent patients could enter at the next dose level at the discretion of the investigators. At other dose levels, a total of three patients had to complete 21 days on study before a decision to dose escalate was made. If a patient withdrew from treatment before day 22 of SP1049C, then this patient was replaced. If DLT was not reached at the seventh dose level of 90 mg m^−2^, further dose increments would be in steps of 20–25% above the prior dose level. If one instance of DLT was observed in three patients, then the dose level would be expanded and an additional three patients treated at that dose level. If one out of six patients experienced DLT, dose escalation would continue. If two out of six patients experienced DLT, three more patients would be treated at a lower dose. This lower dose level would be defined as the MTD unless two out of six patients at that dose level developed DLT, in which case further dose reduction would be made. Patients were assigned to a dose level at the time of enrolment. Intrapatient dose escalation was not permitted.

### Treatment delay or dose modification

A treatment delay of up to 3 weeks was allowed for resolution of DRT. If a patient who had shown clinical benefit from SP1049C developed a DLT, SP1049C could be continued at a lower dose level, up to a maximum of six cycles (in total), at the discretion of the Investigator.

Patients could be withdrawn from the study for any of the following reasons: progressive disease; unacceptable toxicity; cardiac ejection fraction <50% or fall of ⩾15% from baseline; serious adverse event requiring discontinuation of study drug treatment; serious violation of the study drug protocol (including persistent patient attendance failure and noncompliance); withdrawal of consent (if a patient had been in the study for less than 22 days, then an additional patient would be recruited to replace them); any unforeseen event or clinical reason which the investigator felt made further treatment inadvisable.

### Pharmacokinetics

With the first dose of SP1049C, venous blood samples were drawn for assay of doxorubicin concentrations as follows: predose, on completion of infusion and at 5, 10, 20 and 40 min, and at 1, 2, 4, 8, 24, 48, 72 and 96 h after the infusion. Samples of venous blood (7–9 ml) were drawn into heparinised sample tubes from a site separate from the drug administration site. Samples were centrifuged within 20 min of collection at 2000 **g** for 20 min and the plasma was transferred into an appropriately labelled polypropylene tube and stored frozen and protected from light at −70°C until analysis. In the absence of sensitive methods for the analysis of free *vs* pluronic-associated doxorubicin, the determination of plasma concentrations of total doxorubicin was performed by reverse-phase high-performance liquid chromatography (HPLC) following extraction of doxorubicin from plasma. Briefly, standard curves of doxorubicin alone and SP1049C were prepared covering 1–500 ng ml^−1^. Two patient plasma samples obtained 10 min postinfusion were used as internal controls in each analysis run. Within run and between run coefficients of variation were <10% and typically less than 5%. The lower limit of detection was 0.5 ng ml^−1^ with lower limit of quantitation of 1 ng ml^−1^. Standards, internal control and patient samples (500 *μ*l) were extracted with 1 ml methanol/50 *μ*l 20% citric acid and assayed by HPLC with fluorescence detection (Bronchud *et al*, 1990).

### Analysis

Adverse events were graded according to CTC Criteria Version 2. Tumour response was defined according to WHO criteria. Pharmacokinetic analysis was performed using ABBOTTBASE pharmacokinetic systems software on plasma concentration time point data for total doxorubicin for each patient. AUC extrapolated to infinity (AUC_0–∞_) of doxorubicin was calculated using the trapezoidal rule for the sum of AUC to the last measured time point and *C*_pred_/*λz*, where *C*_pred_ is the predicted concentration at the last measured time point and the terminal elimination rate constant (*λz*) is derived from the final log linear phase of the concentration time curve using least-squares regression analysis with visual inspection of the data to determine the appropriate number of data points to include in the calculation of *λz*, and the goodness of fit was assessed by Akaike's information criteria. Each patient's data was analysed by nonlinear least-squares regression analysis to determine individual pharmacokinetic parameters.

## RESULTS

### Patient and tumour characteristics

A total of 26 patients were enrolled into the study and the baseline demographics are shown in Table 1

**Table 1:** Patient and tumour characteristics

No. of patients	26
Male/female	19/7
Median age, years (range)	52 (28–74)
	
*Performance status (Karnofsky)*	
100	2
80/90	24
	
*Primary tumour site*	
Colorectal	5
Oesophagus	5
Soft-tissue sarcoma	4
Lung	3
Mesothelioma	2
Others	7^a^
	
*Prior treatment*	
Surgery	26
Radiotherapy	6
Hormonal/biological	6
Chemotherapy	25
	
*Prior chemotherapy*	
Median number of regimens (range)	2 (1–4)
Previous anthracycline treatment	9

a Ewing's sarcoma (one), ovary (one), kidney (one), cholangiocarcinoma (one), liver (one), neuroblastoma (one), unknown primary (one).

. The following dose levels were evaluated: 5, 10, 20, 35, 50, 70 and 90 mg m^−2^. At the time of analysis, all patients had completed SP1049C treatment. In all, 78 courses of treatment were administered at seven dose levels. At the first three dose levels, one patient was treated per dose level as no toxicity greater than grade 1 was observed. At subsequent dose levels, cohorts of at least three patients were treated. Dose levels were expanded when DLT was observed. A total of seven patients were treated at 35 mg m^−2^ (including one patient who died before day 22 of cycle 1), six patients at 50 mg m^−2^, seven patients at 70 mg m^−2^ (including one patient withdrawn before day 22 of cycle 1) and three patients at 90 mg m^−2^. In total, five patients received six cycles of treatment; 20 stopped earlier because of disease progression or treatment-related toxicity; one patient requested to discontinue treatment.

### Toxicity

All 26 patients were evaluable for toxicity. SP1049C showed a predictable spectrum of toxicities at doses of 35 mg m^−2^ and above. These included reversible, dose-related leucopenia, neutropenia and stomatitis. In addition nausea/vomiting, fatigue and alopecia were common drug-related adverse events. Four patients had grade 2 decrease in LVEF. Overall, the spectrum of adverse events was similar to that seen with conventional doxorubicin. No plantar palmar erythrodysthesia was seen in any patient.

Haematological toxicity for each dose level is shown in Table 2

**Table 2:** Haematological toxicities (worst grade per patient)

**Adverse event**	**Dose level (mg m^−2^)**	**Grade 0**	**Grade 1**	**Grade 2**	**Grade 3**	**Grade 4**
Neutrophils	5	1	0	0	0	0
	10	1	0	0	0	0
	20	1	0	0	0	0
	35	4	1	1	1	0
	50	1	2	1	1	1
	70	1	0	2	0	4
	90	0	0	0	0	3
						
Leucocytes (WBC)	5	1	0	0	0	0
	10	1	0	0	0	0
	20	0	1	0	0	0
	35	4	1	1	1	0
	50	2	1	1	1	1
	70	1	1	1	2	2
	90	0	0	0	1	2
						
Haemoglobin	5	1	0	0	0	0
	10	1	0	0	0	0
	20	0	1	0	0	0
	35	1	3	2	1	0
	50	1	2	3	0	0
	70	2	1	4	0	0
	90	0	1	1	1	0
						
Platelets	5	1	0	0	0	0
	10	1	0	0	0	0
	20	1	0	0	0	0
	35	5	2	0	0	0
	50	3	2	0	1	0
	70	4	1	1	1	0
	90	1	1	1	0	0

. The dose-limiting toxicity comprised myelosuppression and DLT was reached at 90 mg m^−2^. The incidence of grade 3 or worse neutropenia at below 35 mg m^−2^ was zero out of three patients, at 35 mg m^−2^ one out of seven patients, at 50 mg m^−2^ two out of sixpatients, at 70 mg m^−2^ four out of seven patients, at 90 mg m^−2^ three out of three patients. Neutropenia was seen at dose levels of 35–90 mg m^−2^ in 17 (65%) of patient with the nadir occurring between days 8 and 15 postinfusion and with recovery to baseline levels by day 22. Thrombocytopenia of grades 1–3 was seen from dose levels 35–90 mg m^−2^ in 10 (38%) patients. Anaemia grades 1–3 was seen from dose levels 20–90 mg m^−2^ in 20 (77%) patients. There was no grade 4 thrombocytopenia or anaemia at any dose level.

SP1049C induced nonhaematological toxicity at each dose level is shown in Table 3

**Table 3:** Nonhaematological toxicities (worst grade per patient)

**Adverse event**	**Dose level (mg m^−2^)**	**Grade 1**	**Grade 2**	**Grade 3**	**Grade 4**
Nausea	5	0	0	0	NA
	10	0	0	0	NA
	20	0	1	0	NA
	35	0	4	0	NA
	50	2	2	1	NA
	70	3	2	0	NA
	90	0	1	0	NA
					
Stomatitis	5	0	0	0	0
	10	0	0	0	0
	20	0	0	0	0
	35	2	1	0	0
	50	1	1	0	0
	70	1	3	1	0
	90	0	1	1	0
					
Vomiting	5	0	0	0	0
	10	0	0	0	0
	20	0	0	0	0
	35	1	1	1	0
	50	3	0	0	0
	70	1	3	0	0
	90	1	1	0	0
					
Reduction in LVEF	5	0	0	0	0
	10	0	0	0	0
	20	0	0	0	0
	35	1	1	0	0
	50	0	0	0	0
	70	1	3	0	0
	90	0	0	0	0
					
Fatigue	5	0	0	0	0
	10	0	0	0	0
	20	0	0	0	0
	35	0	1	0	0
	50	0	2	1	0
	70	2	4	0	0
	90	2	1	0	0
					
Alopecia	5	0	0	—	—
	10	0	0	—	—
	20	0	0	—	—
	35	2	1	—	—
	50	3	1	—	—
	70	1	4	—	—
	90	2	0	—	—
					
Bilirubin	5	0	0	0	0
	10	0	0	0	0
	20	0	0	0	0
	35	0	0	0	0
	50	1	0	0	0
	70	0	1	0	0
	90	1	0	0	0
					
AST/ALT	5	0	0	0	0
	10	0	0	0	0
	20	0	0	0	0
	35	1	0	0	0
	50	1	1	0	0
	70	1	0	0	0
	90	1	1	0	0

NA=not applicable. ALT=alanine aminotransferase; AST=aspartate aminotransferase; LVEF=left ventricular ejection fraction.

.

#### Cardiac

Four patients had a grade 2 fall (>20%) in their LVEF. The total cumulative dose of anthracyclines (including previous therapy) for these patients was as follows: 320 mg m^−2^ (SP1049C, doxorubicin and epirubicin), 210 mg m^−2^ (SP1049C only), 440 mg m^−2^ (SP1049C and doxorubicin) and 870 mg m^−2^ (SP1049C and doxorubicin). In three of the four patients, there were no changes in ECGs or clinical signs of cardiac impairment. One patient who had received a total of 870 mg m^−2^, who had normal LVEF during SP1049C therapy and had an LVEF at the end of the study of 29%, died subsequent to trial completion during an episode of sepsis. The impaired LVEF from SP1049C and prior anthracycline therapy was considered to have contributed to the death.

#### Gastrointestinal

At doses of >20 mg m^−2^, anorexia, nausea and vomiting were observed although grade 3 or 4 gastrointestinal toxicity was uncommon. Nausea and vomiting responded well to standard antiemetics. Grade 1–3 stomatitis was experienced by 12 (46%) of patients at dose levels 35–90 mg m^−2^, with grade 3 only at the highest two dose levels. At dose levels of 35 mg m^−2^ and above, treatment-related fatigue and alopecia were seen.

### Efficacy

A total of 21 patients (81%) were evaluable for response. No patients had a complete or partial response. One patient with oesophageal carcinoma and liver metastases (previous progression on three cycles of cisplatin 80 mg m^−2^ +5 fluorouracil 1 g m^−2^ days 1–4) treated at 70 mg m^−2^ had a rapid symptomatic improvement in tumour-related dysphagia and pain. During treatment, tumour shrinkage occurred and the CT scan after six cycles showed a partial response, with a complete response in the liver metastases. However, the response was not confirmed 4 weeks later. A second patient with Ewing's sarcoma and lung metastases (previous doxorubicin-containing IVAD with partial response) was treated with 35 mg m^−2^ for five cycles*.* A chest X-ray indicated a partial response after five cycles, but the patient declined follow-up and the partial response could not therefore be confirmed. A third patient with carcinosarcoma of the uterus, ovary and peritoneum (previous progression with both carboplatin single agent and carboplatin-ifosfamide) was treated with SP1049C at 35 mg m^−2^ and had a 28% reduction in measurable disease and a 50% reduction in CA125 level. However, after six cycles of SP1049C, both the tumour dimensions and CA125 had increased slightly. Eight (31%) patients had stable disease with a median time to progression of 17.5 weeks (range 9–24 weeks). A total of 13 (50%) patients had progressive disease. At the time of analysis, 54% of the patients had died. Two of these occurred within 4 weeks of the drug being administered. One patient with oesophageal carcinoma and liver metastases received one cycle of SP1049C at 35 mg m^−2^ but developed total dysphagia 12 days after this and subsequently died of progressive disease. A second patient with adenocarcinoma of the lung received one cycle of SP1049C at 70 mg m^−2^ and 4 days afterwards died of progressive disease.

### Pharmacokinetics

In all, 25 (96%) patients were evaluable for pharmacokinetics. One patient had poor venous access precluding sampling. The plasma pharmacokinetic profile of SP1049C was best described by a three-compartment model with an initial ‘distributional’ phase (mean half-life of 6.6 min), an intermediate phase (mean half-life of 2.83 h) and an ‘elimination’ phase (mean half-life 48.8 h) (Table 4

**Table 4:** Plasma pharmacokinetics of SP1049C doxorubicin

**Patient number**	**Dose (mg m^−2^)**	**Dose (mg)**	**AUC (h *μ*g ml^−1^)**	**Vc (l)**	**Cl (l h^−1^)**	***T*_1/2_ *α* (h)**	***T*_1/2_ *β* (h)**	***T*_1/2_ *γ* (h)**
1	5	10.2	0.10	1.27	0.60	0.110	2.02	25.20
2	10	17.0	0.16	1.81	0.46	0.073	1.76	NA
3	20	33.0	0.38	12.20	84.2	0.083	0.25	31.10
4	35	50.0	2.19	22.70	34.4	0.114	1.96	65.50
6	35	53.0	2.02	26.40	30.2	0.160	3.15	68.00
7	35	55.0	1.44	36.90	40.8	0.157	4.76	59.10
8	35	77.0	1.13	56.90	81.4	0.162	9.55	97.00
9	35	66.0	2.15	35.20	33.1	0.142	6.82	98.70
10	35	60.0	1.37	8.48	41.7	0.071	0.83	25.90
11	50	108.0	2.19	12.70	44.5	0.073	0.69	27.10
12	50	84.0	1.58	55.60	48.6	0.129	1.89	64.10
13	50	121.5	1.65	15.10	63.3	0.069	3.88	47.60
14	50	85.0	2.16	8.51	34.9	0.065	0.89	25.40
15	50	95.0	1.54	50.40	78.7	0.176	6.05	102.00
16	50	95.0	1.93	10.60	47.3	0.084	1.24	35.20
17	70	140.0	4.08	35.90	65.2	0.132	1.07	42.00
18	70	120.0	3.21	10.80	37.7	0.086	2.96	31.50
19	70	134.0	4.20	5.85	29.0	0.089	2.79	31.00
20	70	123.0	2.19	25.00	67.1	0.093	3.05	32.70
21	90	167.0	3.70	39.30	56.4	0.183	4.94	65.50
22	90	185.0	2.91	8.04	46.2	0.062	0.73	22.30
23	90	189.0	4.29	45.40	56.8	0.105	2.96	49.50
24	70	125.0	1.62	18.30	71.4	0.089	1.73	21.30
25	70	154.0	2.95	49.40	87.4	0.108	2.83	39.20
26	70	120.0	2.09	24.10	56.8	0.082	1.91	56.50

**Figure 2:**
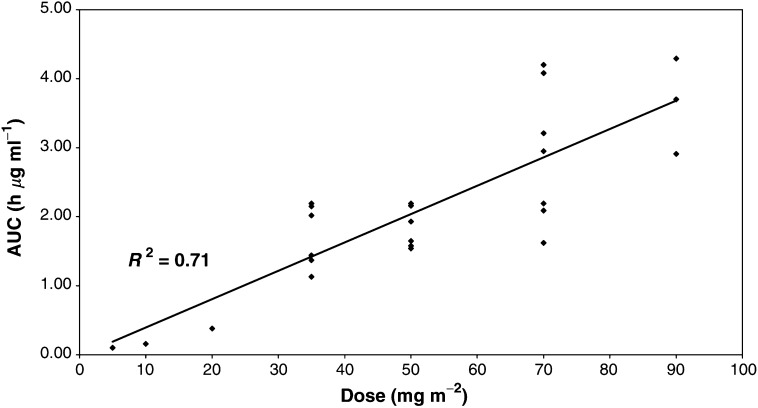
AUC *vs* dose level.

AUC=area under the curves; Vc=Volume (central); Cl=Clearance.

). These values are similar to those reported for conventional doxorubicin although the terminal half-life for conventional doxorubicin in most reports has been shorter at circa 30 h (Eksborg, 1989). The AUC_(0–∞)_ of doxorubicin following SP1049C increased linearly (*r*^2^=0.71) with increasing dose for the dose range of the study (Figure 2

).

## DISCUSSION

Several approaches have been used to try to improve the therapeutic efficacy and toxicity profile of doxorubicin including the development of anthracycline analogues and the incorporation of anthracyclines into drug delivery vehicles. Liposomal drug delivery systems with doxorubicin have been extensively investigated in relapsed ovarian cancer (Muggia *et al*, 1997), breast cancer (Ranson *et al*, 1997) and others (Law *et al*, 1994; Schwartz and Caspar, 1995; Ellerhorst *et al*, 1999; Harrington *et al*, 2001; Judson *et al*, 2001). A number of trials have been performed with polymer–doxorubicin conjugate where there is covalent linkage of polymer to doxorubicin. Initial clinical experience of PK1, a covalent doxorubicin–polymer conjugate, demonstrated anticancer activity and a reduction in doxorubicin dose-limiting toxicities (Vasey *et al*, 1999).

For the covalently linked polymer formulation of doxorubicin, PK1 (mw 30 000), the phase I trial MTD of 320 mg m^−2^ on a 3 weekly regimen is much higher than that for doxorubicin or for SP1049C (Vasey *et al*, 1999). This was predicted by preclinical toxicology of PK1 which demonstrated an rodent LD_10_ of approximately 150 mg m^−2^ (Duncan *et al*, 1998) compared to circa 50 mg m^−2^ for doxorubicin and SP1049C (Alakhov *et al*, 1999). For the liposomal doxorubicin formulation TLC D-99, the MTD in preclinical toxicology in rodents of approximately 100 mg m^−2^ translated well into the clinic where a clinical MTD of 75–90 mg m^−2^ was seen (Lohri *et al*, 1991; Cowens *et al*, 1993; Swenson *et al*, 1998). The clinical use of a pegylated liposomal formulation of doxorubicin (Caelyx/Doxil) was associated with dose-limiting palmar plantar erythrodysthesia when administered at 3 weekly dosing at ⩾45 mg m^−2^ (Ranson *et al*, 1997). This cutaneous toxicity was seen during preclinical evaluation but was not fully predicted by short-term toxicology studies (Working, 1998).

SP1049C is a distinct and novel approach in which doxorubicin is incorporated into micelles with two nonionic copolymers, Pluronic L61 and Pluronic F127. Of particular note is the evidence of antitumour activity of SP1049C in doxorubicin-resistant tumours (Batrakova *et al*, 1996) and in multiple drug resistance (MDR) cells (Venne *et al*, 1996; Alakhov *et al*, 1999). There appear to be several potential mechanisms, including inhibition of drug efflux transporters, abolishment of drug sequestration in acidic compartments, inhibition of the glutathione (GSH)/glutathione (GST) detoxification system and reduction of ATP selectively in MDR cells (Venne *et al*, 1996; Batrakova *et al*, 2001; Kabanov *et al*, 2002). In preclinical models, *in vivo* SP1049C was found to be superior to convential doxorubicin in all nine models tested.

This is the first phase I study with SP1049C. SP1049C was evaluated at seven dose levels between 5–90 mg m^−2^ (all doses expressed as doxorubicin equivalent). Single patient cohorts were evaluated at 5, 10 and 20 mg m^−2^ and these dose levels were not associated with any significant drug-related toxicity. SP1049C exhibited a predictable toxicity spectrum in line with other anthracyclines at doses of 35 mg m^−2^ and above. Dose-related myelosuppression was seen and DLT (neutropenic sepsis) was reached at 90 mg m^−2^. The MTD of SP1049C defined in this study was 70 mg m^−2^. Neutropenia occurred with a nadir between day 8 and 15 post dose, with recovery to baseline levels by day 22. Thrombocytopenia was less pronounced and not dose limiting. As is common with cytotoxic chemotherapy in patients with advanced cancer who have received prior therapy, mild anaemia was common. Nonhaematological toxicity included transient lethargy (similar to that seen with other cytotoxic chemotherapeutic agents in patients with advanced cancer), stomatitis and reversible alopecia. No skin toxicity or palmar plantar erythrodysthesia was seen. Nausea and vomiting was easily controlled with standard antiemetics.

Given the activity of SP1049C in anthracycline-resistant tumours in animal models, the design of this trial allowed patients to have received prior anthracycline therapy. It was required that patients have a baseline LVEF of 55% or more and that they have LVEF reassessments during therapy. Four patients, three of whom had received prior anthracyclines, had a fall in LVEF of CTC Grade 2 during SP1049C. In three cases, no clinical signs of cardiac decompensation or cardiac failure were encountered. However, in one patient the fall in cardiac ejection fraction was felt to contribute to death during an episode of sepsis after trial completion. The cardiac safety of SP1049C cannot be accurately delineated in a phase I study such as this because of the small number of patients studied, the marked hetererogeneity in prior treatment and the variable extent of SP1049C exposure. There remains a need for the monitoring of cardiac safety during phase II and III trials. The pharmacokinetic profile was similar to that described previously with conventional doxorubicin but with a slower terminal clearance.

Evidence of antitumour activity following SP1049C was seen in several patients with advanced solid tumours (Ewing's sarcoma, carcinosarcoma, and oesophageal adenocarcinoma). These three patients had received prior therapy to which one patient had responded but the other two had not. Given the promising preclinical data in anthracycline-resistant tumour settings and the data from this phase I trial, phase II trials of SP1049C at 70 mg m^−2^ in selected advanced cancers are now required to further define its antitumour efficacy and safety in larger cohorts of patients. Phase II trials are currently underway in metastatic oesophageal carcinoma and soft-tissue sarcoma.
